# A Biological Approach to the Synthesis of Silver Nanoparticles with *Streptomyces* sp JAR1 and its Antimicrobial Activity

**DOI:** 10.3797/scipharm.1302-02

**Published:** 2013-03-28

**Authors:** Ritika Chauhan, Abhishek Kumar, Jayanthi Abraham

**Affiliations:** School of Biosciences and Technology, VIT University, Vellore, Tamil Nadu, India.

**Keywords:** *Streptomyces* sp JAR1, Metal nanoparticles, Synergistic effect, Antibiotics, XRD pattern

## Abstract

The biological approach to synthesize metal nanoparticles is an important aspect of current nanotechnology research. Silver nanoparticles have been well-known for their inhibitory and antimicrobial effects. The ever-increasing antibiotic resistance in pathogenic and opportunistic microorganisms is a major threat to the health care industry. In the present investigation, silver nanoparticles have been successfully biosynthesized by *Streptomyces* sp JAR1. Biosynthesized silver nanoparticles were characterized by means of several analytical techniques including a UV-Visible spectrophotometer, Fourier transform infrared spectroscopy, X-ray diffraction pattern analysis, and atomic force microscopy. An evaluation of the antimicrobial activity of silver nanoparticles (AgNPs) was carried out against clinically important pathogenic microorganisms. The metal nanoparticles were also evaluated for their combined effects with antibiotics against the clinical pathogens. The antibacterial activities of the antibiotics increased in the presence of the biologically synthesized AgNPs against the clinically important pathogens. The highest enhancing effect was observed for erythromycin against the test pathogens.

## Introduction

The synthesis of metallic nanoparticles is an active and pronounced area of research in nanotechnology. Metal nanoparticles have been intensively studied in the past decade. Nanosized materials have been an important subject in basic and applied sciences because of their unique optical, thermal, electrical, chemical, and physical properties that are due to a combination of the large proportion of high-energy surface atoms compared to the bulk solid [1]. The development of novel applications in the pharmaceutical field with metallic nanoparticles makes it an attractive alternative to antibiotics. Metallic nano-particles have been examined for their ability to reduce microbial infections in the skin [2] and burn wounds [3], and also to prevent bacterial colonization on various surface devices such as catheters [4] and prostheses [5]. In earlier days, silver was used extensively to saturate bandages so as to restrict bacterial growth in injured skin [6, 7] but toxic effects of silver nanoparticles have been reported in mammalian cells, including an alteration in the normal function of mitochondria, an increase in membrane permeability, and the generation of reactive oxygen species [8, 9]. Several chemical methods have been developed for the synthesis of silver nanoparticles including chemical reduction [10], aqueous solution chemical reduction [11], nonaqueous chemical reduction [12], the template method [13], electrochemical reduction [14], ultrasonic-assisted reduction, photo-induced or photo-catalytic reduction [15], microwave assisted synthesis [16], irradiation reduction [17], the microemulsion method [18], and the biochemical method etc. But these chemical methods have been reported along with various drawbacks of many problems including the use of toxic solvents, generation of hazardous by-products, and high energy consumption, which pose potential risks to human health and to the environment. Currently, there is a growing need to develop an environmentally friendly nanoparticle synthesis that does not use toxic chemicals in the process of its synthesis [19]. The microbial-mediated biological synthesis of metallic nanoparticles has recently been recognized as a promising source for mining nanomaterials [20]. The microbial recovery of precious metals with the formation of their nanoparticles is a green alternative to the conventional method. Biosynthesis of silver nanoparticles using bacteria, fungi, and plants [16, 17] are already well-documented. However, the exploration of actinomycetes has recently gained interest for the efficient biological synthesis of metallic nanoparticles [20]. In the present investigation, we examine and characterize the extracellular biosynthesis of AgNPs from the extracellular components of *Streptomyces* sp JAR1. The physical properties of biosynthesized AgNPs were studied through sophisticated analytical instrumentation including the UV-visible absorption spectrophotometer, Fourier transform infrared spectroscopy (FT-IR), X-ray diffraction pattern analysis (XRD), and atomic force microscopy (AFM). Furthermore, the antimicrobial properties and combined effects with antibiotics have been studied to ensure the contribution of biologically synthesized silver nanoparticles to nanomedicine.

## Results and Discussion

### Isolation and characterization of the strain

**Fig. 1.**
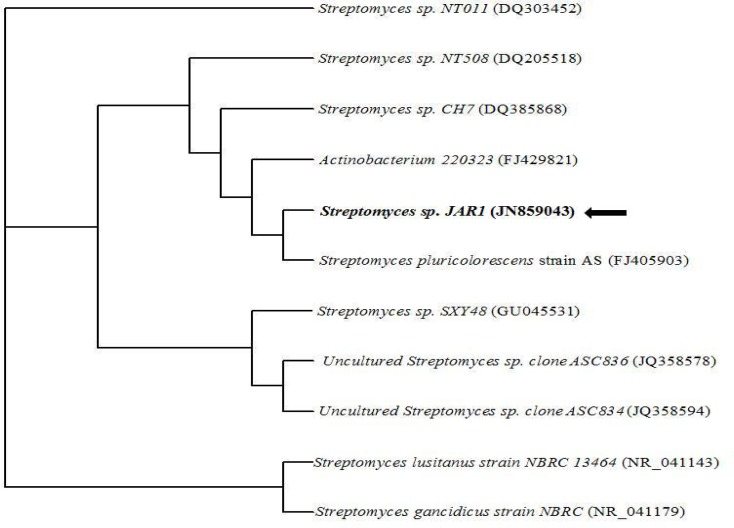
Phylogenetic relationship based on the 16S rRNA gene nucleotide sequences between the *Streptomyces* sp. JAR1 and reference sequences retrieved from the NCBI Gen Bank constructed through the neighbor joining method.

The pure colonies of the isolated strain were obtained and the isolate was characterized as *Streptomyces* sp JAR1 based on the molecular characterization through the 16S rRNA sequencing studies as shown in Figure 1. The morphological and cultural characteristics of *Streptomyces* sp JAR1 were performed according to [21] protocol and are presented in Table 1.

### Microbial synthesis of AgNPs

Silver nanoparticles were biologically synthesized by the culture supernatant *Streptomyces* sp JAR1. The appearance and color change from brown to yellowish-brown in the silver nitrate-treated flask indicated the formation of silver nanoparticles, whereas no color change was observed in the culture supernatant without silver nitrate.

### UV-Spectrophotometer study

**Fig. 2.**
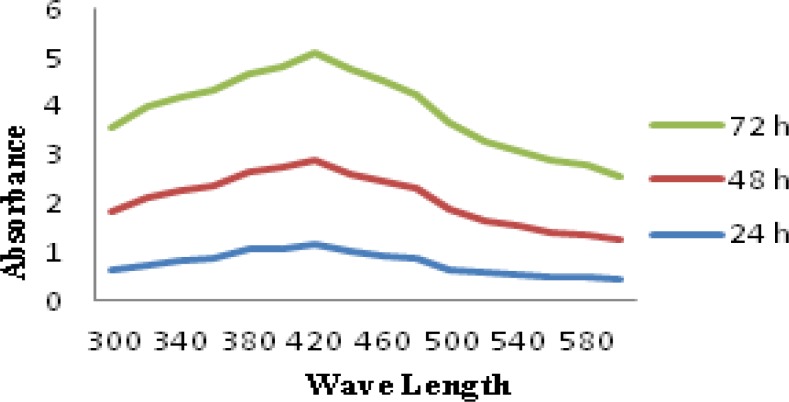
UV-Visible absorption spectrum of AgNPs synthesized by extracellular components of *Streptomyces* sp JAR1.

The formation and stability of the reduced silver nanoparticles in the colloidal solution was monitored by a UV–vis spectrophotometer [22]. The intensity of the biosynthesized AgNPs showed increased absorbance in various time intervals of 24h, 48h, and 72h. A strong, broad peak was observed between 420nm and 425 nm, corresponding to the surface plasmon resonance (SPR) of the silver nanoparticles. The increase in the intensity may have been due to the excitation of SPR and the reduction of AgNO_3_ where the increasing time interval of 24h, 48h, and 72h is well-documented by various metal nanoparticles [23]. The synthesis of metal nanoparticles depends on the nitrate reductase enzyme present in the microbes. The mechanism of the biosynthesized nanoparticles involves the reduction of silver ions by the electron shuttle enzymatic metal reduction process. NADH and NADH-dependent enzymes are important factors in the biosynthesis of metal nanoparticles [24]. The microbes are known to secrete the cofactor NADH, and NADH-dependent enzymes like nitrate reductase might be responsible for the bioreduction of metal ions and the subsequent formation of silver nanoparticles [22]. The UV-Vis absorption spectrum is regarded as a potential application for the subsequent processing of silver nanoparticles, where the absorption peak at 420–425nm indicates the successful synthesis of AgNPs after 72h. Figure 2 represents the UV-spectrum of the biosynthesized silver and nanoparticles.

### FT-IR Analysis

**Fig. 3.**
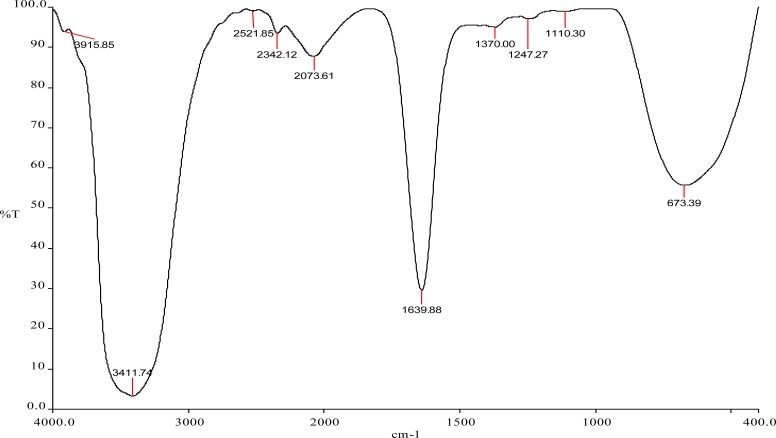
FT-IR spectrum of AgNPs synthesized by *Streptomyces* sp JAR1.

The FT-IR spectrum was recorded from the potassium iodide pellet with the biosynthesized silver nanoparticle formed after 72h of incubation with *Streptomyces* sp JAR1. Figure 3 shows the amide linkages between the amino acid residues in proteins giving rise to the well-known signatures in the infrared region of the electromagnetic spectrum. The bands observed at 3411 cm^−1^, which fall near 3280cm^−1^, were assigned to the stretching vibrations of the primary amines, while the corresponding bending vibrations were seen at 1639 cm^−1^. The two small bands observed at 1370 cm^−1^ and 1110 cm^−1^ represent the C-N stretching vibrations of aromatic and aliphatic amines, respectively. The bands observed in the FT-IR of the biosynthesized AgNPs using *Streptomyces* sp JAR1 confirm the presence of proteins. The proteins present in the AgNPs sample are able to bind nanoparticles either through free amine groups or cysteine residues in the proteins [25] through electrostatic attraction of negatively charged carboxylate groups [26] present in the enzyme produced by Streptomyces sp JAR1. The formation and stability of the biosynthesized AgNPs by proteins is a possibility [26].

### X-ray diffraction analysis

**Fig. 4.**
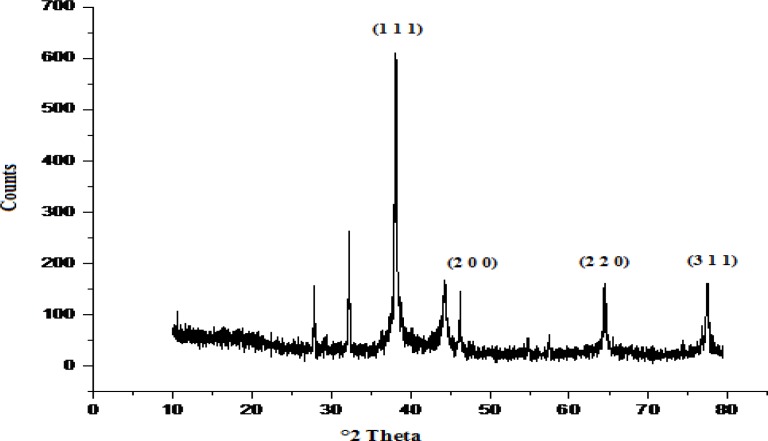
XRD pattern of biosynthesized AgNPs by extracellular components of *Streptomyces* sp JAR1

The XRD pattern of the silver nitratetreated sample shows four intense peaks in the 2θ spectrum ranging from 30 to 80. The exact nature of the biosynthesized silver nanoparticles formed can be deduced from the XRD spectrum of the sample. The characteristic XRD peaks 2θ at 38°, 45°, and 65° correspond to the (1 1 1), (2 0 0), and (2 2 0) planes for silver, respectively. The diffraction peaks indexed at (1 1 1), (2 0 0), and (2 2 0) representing crystalline structures were well-documented for silver previously by various researchers [27], [22]. The strong, narrow diffraction peaks shown in Figure 4 indicate that the product has a crystalline structure.

### Atomic force microscopy

**Fig. 5.**
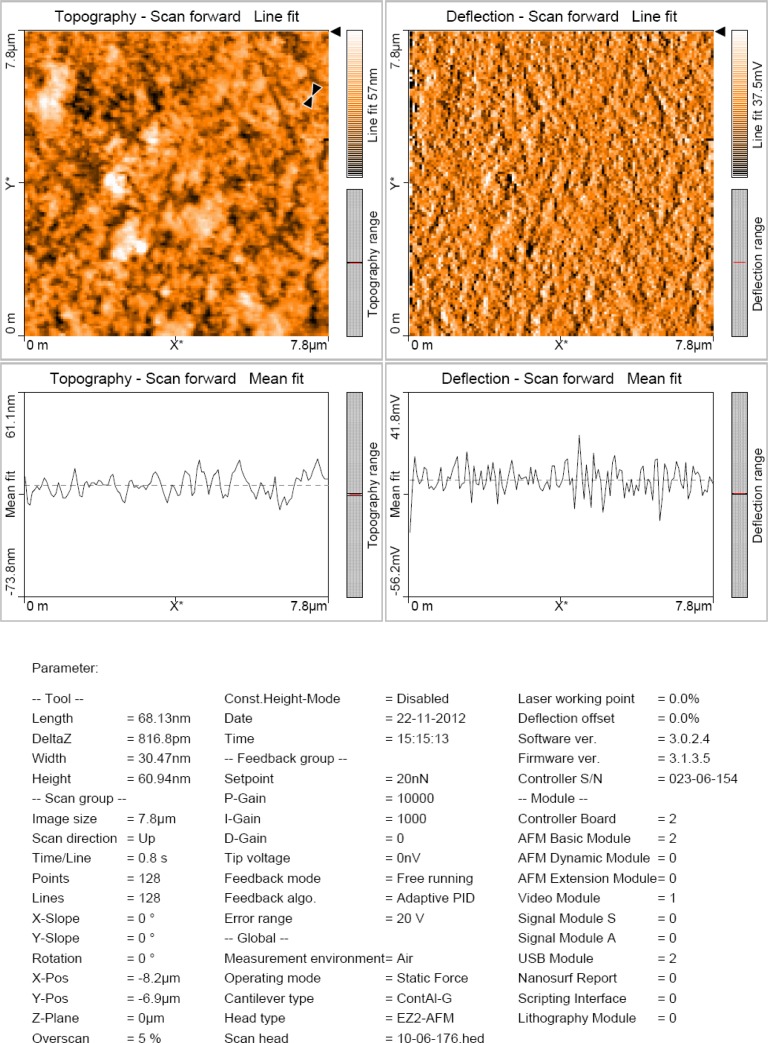
Atomic force Microscopic image of biologically synthesized AgNPs showing topographical characteristics and particle size of 68.13nm of synthesized AgNPs.

An atomic force microscopy was performed to identify the topological appearance, and the size of the biosynthesized silver nanoparticles was found to be 68.13 nm. The AFM images were also used for the analysis of the fractal behavior of the deposited and annealed films. Porosity, roughness, and fractal dimension were evaluated by analyzing the AFM images. The particles size analysis study of the silver nanoparticles gives the average size of 68.13 nm as shown in Figure 5.

### Antimicrobial activity of AgNPs

The biologically synthesized AgNPs inhibited different pathogenic microorganisms. The resulting zones of inhibition formed were mainly due to the destabilization of the outer membrane, collapse of the plasma membrane, and depletion of intracellular ATP by the silver nanoparticles. *Escherichia coli*, *Pseudomonas aeruginosa*, *Salmonella* sp*, Staphylococcus aureus, Ganoderma* sp JAS4*, Scedosporium* sp JAS1*, Fusarium* sp, *and Candida tropicalis* were effectively inhibited by the silver nanoparticles as shown in Table 2 and Table 3. According to [28], the mechanism behind the bactericidal effect of the silver nanoparticles against bacteria is not well known. It has been proposed that AgNPs act similarly to the antimicrobial agents used for the treatment of bacterial infection by different mechanisms. There are three different mechanisms explained by [29]. Firstly, Ag NPs attach to the surface of the cell membrane and disturb its power functions, such as permeability and respiration [30]. The binding of the particles to the bacteria depends on the interaction of the surface area available. With a smaller particle size, a large surface area will have a stronger bactericidal effect. Secondly, Ag NPs are able to penetrate the bacteria by possibly interacting with sulfur- and phosphorus-containing compounds such as DNA and cause further damage [31]. Thirdly, the silver nanoparticles release silver ions, which contribute to the bactericidal effect [32]. The mechanism of inhibition by silver ions on microorganisms is partially known. It is believed that DNA loses its replication ability and cellular proteins become inactivated upon silver ion treatment [33, 34]. Furthermore, higher concentrations of Ag^+^ ions have been shown to interact with cytoplasmic components and nucleic acids [32, 35].

### Synergistic effect of AgNPs

The combination of antibiotics with AgNPs against Gram-positive and Gram-negative bacteria and fungal test strains offers a valuable contribution to nanomedicine. The antibacterial activities of tigecycline, vancomycin, erythromycin, and ofloxacin increased in the presence of AgNPs against the test strains. The synergistic effect of silver nanoparticles represents the highest percentage of increase in inhibition, which was found against vancomycin (30mcg/disk), followed by erythromycin (15mcg/disk), ofloxacin (5mcg/disk), and tigecycline (15mcg/disk) against all test strains as shown in Table 3. The diameter of the zone of inhibition increased with tigecycline, vancomycin, erythromycin, and ofloxacin in the presence of the metallic nanoparticles against the test strains. The bonding reaction between the antibiotic and nanoparticles enhanced the inhibition effect against the test organisms. The antibiotic molecules contain many active groups such as hydroxyl and amide groups, which react easily with nanosilver by chelation, which helps in effective inhibition [27]. The biosynthetic methods have been recognized as an alternative to chemical and physical synthesis, as this biosynthetic method is economical, eco-friendly, and cost-effective. The present work exhibited an efficient and low-cost biological approach to synthesize the metal nanoparticles and provided helpful insight into the development of new antimicrobial agents with the synergistic enhancement of the antibacterial mechanism against pathogenic microorganisms.

## Experimental

### Collection of soil sample

Soil samples were collected from the Kodaikanal hill station, Tamil Nadu, India in the month of September 2011.

### Isolation of Streptomyces sp JAR1

Soil samples were serially diluted and spread on starch-casein-nitrate agar plates. The plates were then incubated at 30°C for 7d.

### Taxanomy and identification of the isolate

The cultural characteristics of the strain *Streptomyces* sp JAR1 were studied through the International Streptomyces Project (ISP) [21]. The genomic DNA was extracted using the Chromus Genomic DNA Isolation Kit (Chromus Biotech Pvt, Ltd., Bangalore, India). The isolated genomic DNA was loaded and run on 1% agarose gel. For amplification of 16srRNA gene, the forward primer (400ng) 5′AGAGTRTGATCMTYGCTWAC-3′ and reverse primer (400ng) 5′-CGYTAMCTTWTTACGRCT-3′, 2.5mM each of dNTPs, 10X Taq polymerase assay buffer and Taq DNA polymerase enzyme were used, keeping the reaction volume at 100μl. The amplification reaction was further followed by the initial denaturation at 94°C for 5 mins, denaturation at 94°C for 30 s, and annealing at 55°C for 30 s leading to final extension at 72°C using Mgcl_2_ with 1.5mM as the final concentration. The amplified product was sequenced with the primer using the ABI 3130 Genetic Analyzer (Chromous Biotech Pvt. Ltd., Bangalore, India). The phylogenetic position of *Streptomyces* sp JAR1 was assessed by performing a nucleotide sequence database search using the BLAST program from the NCBI GenBank. The nucleotide sequencing result was submitted to the GenBank National Centre for Biotechnology Information (NCBI) and the accession number obtained was JN859043.

### Microbial Synthesis of AgNPs

The *Streptomyces* sp JAR1 culture was freshly inoculated on sterile ISP-2 medium and the flasks were incubated at 28°C and 200rpm at pH 7.2 for 96 h GHH. After the 96h of incubation, the culture was centrifuged at 4000rpm for 30 min and the supernatant was used for the biosynthesis of AgNPs. The deionized water was used as a solvent for the biological synthesis of AgNPs. The supernatant of *Streptomyces* sp JAR1 was collected and was added separately as 1% (v/v) to the reaction vessel containing silver nitrate (1mM concentration) and incubated on an orbital shaker under dark conditions for 96 h at 30°C [22].

### UV–Visible spectral analysis

The color change from brown to yellow portrays the bioreduction of the Ag^+^ ions in the medium containing silver nitrate. The extracellular components of the *Streptomyces* sp JAR1 culture reduced the Ag^+^ ions in the medium. The absorption spectrum of this solution was recorded using a UV–Visible spectrophotometer (HITACHI, Model U-2800 spectrophotometer) from 300nm to 600nm at 24h, 48h, and 72h [22]. The biosynthesized silver nanoparticles were observed showing a particular plasmon resonance at 420nm. The observation of this peak was measured at regular intervals to determine their stability. In this analysis, the supernatant of the strain *Streptomyces* sp JAR1 and silver nitrate solution were used separately as controls.

### FT-IR analysis

The biosynthesized silver nanoparticles were mixed with potassium bromide powder to form a pellet. The pellet was further analyzed using the Fourier transform infrared spectrophotometer (FT-IR) using the diffuse reflectance accessory [23].

### X-ray diffraction analysis

The biologically synthesized silver nanoparticles using *Streptomyces* sp JAR1 were dried, powdered, and used for XRD analysis. The XRD patterns were recorded using the X-ray powder diffractometer (Model-D8 Advance, made in BRUKER Germany) at 40 kV/20m using the continuous scanning 2θ mode to check the formation, purity, and stability of the silver nanoparticles [38].

### Atomic Force Microscopy

The topography of silver nanoparticles was studied using AFM (Model-Nanosurf easyscan 2 AFM, made in Switzerland) working in the contact mode. The size of the nano-objects was measured with atomic resolution [39]. A thin film of the sample was prepared on a glass slide by dropping 100 μL of the sample onto the slide, and was allowed to dry for 5 min. Topographical images were obtained in non-contact mode using silicon nitride tips at a resonance frequency of 218 kHz.

### Antimicrobial activity of AgNPs

The antimicrobial activity of the biologically synthesized AgNPs against pathogenic organisms such as Gram-positive (*Enterococcus faecalis and Staphylococcus aureus*), Gram-negative (*Escherichia coli, Salmonella typhimurium, Shigella sp, Proteus mirabilis, Klebsiella pneumoniae, Pseudomonas aeruginosa*), and fungal strains (*Candida tropicalis*, *Fusarium* sp, *Scedosporium* sp JAS1, *Ganoderma* sp JAS4, *Aspergillus terreus* strain JAS1) was measured using the well-diffusion method. The clinical pathogens were procured from the Microbial Biotechnology Lab, SBST, VIT University, Vellore, India. Pure cultures of the bacteria were grown in Mueller-Hinton broth (Hi-media, Mumbai, India) at 37°C on a rotary shaker at 200 rpm. Wells of 6mm in diameter were punctured in the Mueller-Hinton agar plates using a gel puncture and each well was inoculated with individual cultures. Then 25μl, 50μl, 75μl, and 100μl of the biosynthesized AgNPs solution was added into each well. After incubation, the diameter of the zone of inhibition was measured. Pure cultures of fungi were maintained in potato dextrose agar (Hi-Media, Mumbai, India) at 30°C for 5d. For antifungal activity, the appropriate fungal test pathogens were seeded in potato dextrose agar (PDA) in petridishes [40]. Paper disks of 6mm in diameter were laid on the inoculated test organism after being soaked with 25μl, 50μl, 75μl, and 100μl of the biosynthesized AgNPs and antimicrobial activity was determined by measuring the zone of inhibition around the disk.

### Synergistic effect of AgNPs

The disk diffusion method was used to investigate the synergistic effect of antibiotics with extracellularly synthesized AgNPs for bactericidal activity against test strains on Muller-Hinton agar plates. In this assay, each standard antibiotic disk was further impregnated with 10 μL of freshly prepared AgNPs [36]. A single colony of each test strain was grown overnight in Muller-Hinton liquid medium on a rotary shaker (200 rpm) at 37°C. The Muller-Hinton agar medium plates were seeded with 100μl of test organisms and the antibiotic disks impregnated with AgNPs were placed onto the agar plates. After incubation at 37°C for 24h, the zones of inhibition were measured.

## Authors’ Statement

### Competing Interests

The authors declare no conflict of interest.

## Figures and Tables

**Tab. 1.** Morphological and cultural characteristics of *Streptomyces* sp JAR1.

**Sr No.**	**Culture Medium**	**Growth**	**Aerial mycelium**	**Substrate mycelium**	**Diffusable pigment**	**Melanoid Pigment**
1	Tryptone-yeast agar medium (ISP-1)	Poor	White	Pale brown	–	–
2	Yeast extract malt-extract agar (ISP-2)	Very Good	Grey	Brown	Dark Brown	+
3	Oatmeal agar (ISP-3)	Good	Grey	None	Brown	–
4	Inorganic salt-starch agar (ISP-4)	Good	Grey	None	–	–
5	Glycerol asparagine agar (ISP-5)	Good	White	Pale yellow	Brown	–
6	Peptone yeast iron agar (ISP-6)	Good	NG	Light brown	–	–
7	Tyrosine agar (ISP-7)	Moderate	Grey	Brown	–	–

**Tab. 2.** Antibacterial activity of extracellularly biosynthesized silver nanoparticles by *Streptomyces* sp JAR1 against Gram-positive and Gram-negative pathogens.

**Sr No.**	**Test organisms**	**Diameter zone of inhibition (mm)**			
		**25μl**	**50μl**	**75μl**	**100μl**
1	*Escherichia. coli*	10.00±0.81	12.33±0.47	12.66±0.47	13.33±0.47
2	*Pseudomonas aeruginosa*	9.66±0.47	11.00±0.81	12.33±0.47	12.66±0.47
3	*Salmonella* sp	10.66±0.47	11.33±0.47	11.66±0.47	12.33±0.94
4	*Staphyloccocus aureus*	8.33±0.47	9.33±0.47	10.66±0.47	11.66±0.47
5	*Enterococcus* sp	7.66±0.47	9.00±0.00	10.33±0.47	11.33±0.47
6	*Klebsiella pneumonia*	–	–	–	–
7	*Shigella* sp	–	–	–	–
8	*Proteus mirabilis*	9.33±0.47	11.00±0.81	11.66±0.47	13.00±0.00

The study was found significant level at <0.05 (p-value).

**Tab. 3.** Zone of inhibition of extracellularly biosynthesized silver nanoparticles against fungal pathogens.

**Sr No.**	**Test organisms**	**Diameter zone of inhibition (mm)**			
		**25μl**	**50μl**	**75μl**	**100μl**
1	*Candida tropicalis*	–	–	–	–
2	*Fusarium* sp	16.00±0.00	17.33±0.47	17.66±0.47	21.66±0.47
3	*Scedosporium* sp. JAS1	–	–	–	–
4	*Ganoderma* sp. JAS4	–	–	–	–
5	*Aspergillus terreus* strain JAS1	11.00±0.81	13.66±0.47	15.00±0.81	16.33±0.47

The study was found significant level at <0.05 (p-value).

**Tab. 3.** Zone of inhibition of combined effects of extracellularly biosynthesized AgNPs with different antibiotics (with and without antibiotics) against Gram-positive and Gram-negative bacteria.

	**Diameter zone of inhibition (mm)**					

**Antibiotic**	**Tigecycline**			**Vancomycin**		
	
**Test Pathogen**	**Ab**	**Ab + NP**	**%**	**Ab**	**Ab + NP**	**%**
*Escherichia coli*	17	20	6.64	14	15	7.14
*Pseudomonas aeruginosa*	19	20	5.26	15	20	33.34
*Salmonella* sp	13	17	30.76	–	30.76	–
*Staphylococcus aureus*	18	20	11.12	17	19	11.16
*Shigella* sp	16	19	18.75	13	14	7.69
*Proteus mirabilis*	15	29	26.67	15	18	20.00
*Enterococci* sp	13	15	15.38	12	15	25
*Klebsiella pneumoniae*	12	20	66.67	17	18	7.89

	**Diameter zone of inhibition (mm)**					

**Antibiotic**	**Erythromycin**			**Ofloxacin**		
	
**Test Pathogen**	**Ab**	**Ab + NP**	**%**	**Ab**	**Ab + NP**	**%**

*Escherichia coli*	19	40	110.5	15	16	6.67
*Pseudomonas aeruginosa*	22	27	22.72	28	29	3.57
*Salmonella* sp	18	22	22.22	28	30	7.13
*Staphylococcus aureus*	30	35	16.66	28	29	3.57
*Shigella* sp	12	19	58.33	17	18	7.89
*Proteus mirabilis*	10	16	60.00	18	20	11.12
*Enterococci* sp	13	17	30.76	30	32	6.76
*Klebsiella pneumoniae*	28	30	7.142	24	39	24.16

Ab (a)…Antibiotic disc; Ab + Np (b)… Antibiotic disc; Over all percentile increase % = 100*(b–a)/a
